# The contrary functions of lncRNA HOTAIR/miR‐17‐5p/PTEN axis and Shenqifuzheng injection on chemosensitivity of gastric cancer cells

**DOI:** 10.1111/jcmm.13970

**Published:** 2018-10-19

**Authors:** Jianguang Jia, Dankai Zhan, Jing Li, Zhixiang Li, Hongbo Li, Jun Qian

**Affiliations:** ^1^ Department of Surgical Oncology The First Affiliated Hospital of Bengbu Medical College Bengbu City China

**Keywords:** chemoresistance, gastric cancer, lncRNA HOTAIR, miR‐17‐5p, PTEN, Shenqifuzheng injection

## Abstract

This study was implemented to figure out whether lncRNA HOTAIR/miR‐17‐5p/PTEN axis played a role that was opposite to Shenqifuzheng (SQFZ) injection in regulating the chemosensitivity of gastric cancer cells. The gastric cancer tissues were gathered and four gastric cancer cell lines were prepared, including BGC‐823, MGC‐803, SGC‐7901, and MKN28. Moreover, cisplatin, adriamycin, mitomycin, and 5‐fluoroura were managed as the chemo‐therapeutics, and SQFZ was prepared as a Chinese medicine. Striking distinctions of HOTAIR, miR‐17‐5p, and PTEN expressions were observed between gastric cancer tissues and para‐carcinoma normal tissues (*P *<* *0.05). MKN28 was associated with the highest resistance to cisplatin, adriamycin, mitomycin, and 5‐fluoroura among all the cell types, and SQFZ significantly improved the MKN28 cells’ sensitivity to the drugs (*P *<* *0.05). The over‐expressed HOTAIR and miR‐17‐5p, as well as under‐expressed PTEN tended to significantly facilitate the viability, EMT process and proliferation of MKN28 cells that were subject to treatment of chemo‐therapies (*P *<* *0.05). SQFZ could amplify the effects of si‐HOTAIR, miR‐17‐5p inhibitor, and pcDNA‐PTEN on boosting the chemosensitivity of gastric cancer cells (*P *<* *0.05). In addition, HOTAIR was also found to directly target miR‐17‐5p, and PTEN appeared to be subject to the modification of HOTAIR and miR‐17‐5p in its acting on the viability, proliferation, EMT process, and apoptosis of gastric cancer cells. The HOTAIR/miR‐17‐5p/PTEN axis could be regarded as the potential treatment targets for gastric cancer, and adjuvant therapy of SQFZ injection could assist in further improving the treatment efficacy of chemo‐therapies for gastric cancer.

## 1 INTRODUCTION

Gastric cancer is ranked second among all the malignancies considering its global morbidity and mortality, and its recognized clinical characteristics are commonly comprised of insidious onset, missed diagnosis, and high recurrence rate.^1^ According to statistics, newly developed gastric cancer cases have already taken up more than 17% of all malignant neoplasms in China, and the fatality rate induced by gastric cancer also accounts for over 20% of the whole.^2^ Moreover, a majority of gastric cancer patients have been at a progressive stage when they were confirmed diagnosis, owing to lacking sensitive biomarkers for early‐stage gastric cancer. For gastric cancer patients at the advanced stage, the operation‐oriented comprehensive therapy was regarded as the optimal choice, yet its causing 5‐year survival rate was limited to barely 20%‐50%.^3^ One explanation for the low survival rate appeared as that misjudging the patients’ sensitivity to certain drugs (ie chemo‐therapies) could delay their appropriate treatments.^4^, ^5^ Thus, figuring out the mechanisms inherent in chemosensitivity of gastric cancer took on high importance.

Notably, lncRNAs were extensively involved with cell differentiation, cellular metabolism and cell proliferation, which enabled their close correlation with onset and progression of diversified diseases.^6^ Especially, their expressions could biologically mark the process of tumor formation and development.^7^ For example, lncRNA HOTAIR, located in the HOXC locus of chromosome 12 with a length of 2.2 kb, was the first ascertained lnc RNA in association with neoplastic metastasis.^8^ Its expression within primary and metastatic thymoma was elevated by up to 2000 times in comparison to that within normal thymus tissues.^9^ Also highly‐expressed HOTAIR were more frequently detected within gastric patients who were with lymphatic metastasis and at an advanced stage of TNM grading.^10^ Hence, HOTAIR was suspected to participate in the etiology of gastric cancer.

Moreover, the specific secondary structure made it possible for lncRNAs to interact with DNAs and RNAs based on the principle of complementary base pairing.^11^ It was previously figured out that HOTAIR could target miR‐17‐5p within human cervical cancer cells.^12^ As far as miR‐17‐5p was concerned, it was more highly expressed within gastric cancer tissues than within para‐carcinoma tissues.^13^, ^14^ Also it was found to gear up angiogenesis and tumor proliferation, as well as to hold up cell apoptosis and cell senescence, which implied that miR‐17‐5p could contribute to enhancive chemo‐resistance.^15^, ^16^, ^17^ In addition, miR‐17‐5p/PTEN axis has also deemed as a pivotal signaling underlying tumor development.^18^, ^19^, ^20^ Abnormal lowly expressed PTEN, an anti‐oncogene featured by phosphatase activity,^21^ was linked with promoted onset and progression of neoplasms (eg gastric cancer).^21^, ^22^, ^23^ However, although HOTAIR, miR‐17‐5p and PTEN have been, respectively, indicated to participate in development and chemosensitivity of gastric cancer, hardly any investigations have been focused on their synthetic contributions.

Furthermore, several traditional Chinese medicines (TCMs) have also been regarded as potent candidates for ameliorating the chemo‐resistance of cancers, yet few investigations have intended to explore this part. For instance, SQFZ injection, also known as ginseng and astragalus righting injection, was composed of extracts from astragalus membranaceus and codonopsis pilosula.^24^ The astragalus membranaceus therein was pharmaceutically analysed as the mixture of astragalin, astragalus flavone, astragalus polysaccharides, small amounts of mineral substances, and it was clinically indicated to improve body immunity and suppress tumor growth.^25^ All in all, HOTAIR/miR‐17‐5p/PTEN axis, and SQFZ injection were, respectively, hypothesized as crucial participants in the oncogenesis and chemosensitivity of gastric cancer. However, they have not been investigated together within one study, therefore, this investigation was aimed to remedy this gap.

## 2 MATERIAL AND METHOD

### 2.1 Collection of tissue samples

From January 2016 to March 2017, we gathered 317 gastric cancer tissues and 317 adjacent non‐tumorous stomach tissues that were freshly frozen and paraffin‐embedded from the First Affiliated Hospital of Bengbu Medical College. All cases were reviewed by the pathologist, and were confirmed as gastric cancer based on histopathological assessment. No local or systemic treatment was performed for gastric cancer patients before the operation. The ethics committee of First Affiliated Hospital of Bengbu Medical College and the First Affiliated Hospital of Bengbu Medical College has authorized this study. All the participants have signed informed consents.

### 2.2 Cell Culture

Four types of gastric cancer cell lines (ie MGC‐803, SGC‐7901, BGC‐823, and MKN28) and normal gastric epithelial cell line (ie GES‐1) were all purchased from Shanghai Institute of Biochemistry and Cell Biology (China). The cell lines were cultured using RPMI‐1640 or DMEM medium (GIBCO‐BRL, Gaithersburg, MD, USA) that have been added with 10% fetal calf serum (FBS), 100 U/mL penicillin, and 100 mg/mL streptomycin (Invitrogen, Carlsbad, CA, USA), and were placed in moist air, and the atmosphere was managed as 37°C, 5% CO_2_ and moist.

### 2.3 Treatment with Shenqi Fuzheng (SQFZ)

SQFZ [National drug standard No.: WS3‐387(Z‐50)‐2003(Z)] was purchased from Livzon pharmaceutical corporation (Guangdong, China), and 10 mL of its concentrated solution was equivalent to 5 g codonopsis pilosula and 5 g crude drugs of astragalus membranaceus. The cells were processed with SQFZ injection with different concentrations of 0, 55, 110, 220 mg/mL, respectively.

### 2.4 RNA extraction and qRT‐PCR analysis

The TRIZOL reagent (Invitrogen, USA) was applied to extract the total RNA from tissues or cultured cells. Spectrophotometry method was taken to measure the purity as well as the concentration of RNA. The extracted RNA was stored at −80°C before RT‐PCR analysis.

For qRT‐PCR, RNAs were reversed into cDNAs with the RT‐PCR kit (Takara, Beijing, China) under conditions of 42°C for 60 minutes, 95°C for 5 minutes, and 4°C for 10 minutes. With usage of Power SYBR Green (Takara, China), the qRT‐PCR conditions for HOTAIR were set as 40 cycles of initial denaturation at 95°C for 30 seconds, denaturation at 95°C for 2 minutes and annealing at 55°C for 1 minute. With respect to miR‐17‐5p and PTEN, their qRT‐PCR conditions were specified as 40 cycles of initial denaturation at 95°C for 30 seconds, denaturation at 95°C for 5 seconds and annealing at 60°C for 30 seconds. GAPDH was utilized as the internal reference for HOTAIR and PTEN, and U6 snoRNA was designated as the internal reference for miR‐17‐5p. The relative expressions of target genes were calculated according to 2^−▵▵Ct^ method. The primers (Table 1) were designed with ABI Primer Express software, and were synthesized by Shanghai Sango Corporation (China).

**Table 1:** List of primers utilized for quantitative real time polymerase chain reaction (qRT‐PCR)

Gene	Primer sequence
miR‐17‐5p	5′‐GCCGCCAAAGTGCTTACA‐3′ (forward)
	5′‐CAGAGCAGGGTCCGAGGTA‐3′ (reverse)
U6	5′‐CGCTTCGGCAGCACATATAC‐3′ (forward)
	5′‐AATATGGAACGCTTCACGA‐3′ (reverse)
LncRNA HOTAIR	5′‐GATCCGCCACATGAACGCCCAGAGATTTTCAAGAGAAATCTCTGGGCGTTCATGTGGTTTTTTG‐3′ (forward)
	5′‐AATTCAAAAAACCACATGAACGCCCAGAGATTTCTCTTGAAAATCTCTGGGCGTTCATGTGGCG‐3′(reverse)
PTEN	5′‐TCCACAAACAGAACAAGATG‐3′ (forward)
	5′‐CTGGTCCTGGTATGAAGAAT‐3′ (reverse)
GAPDH	5′‐GGGAGCCAAAAGGGTCAT‐3′ (forward)
	5′‐GAGTCCTTCCACGATACCAA‐3′ (reverse)

### 2.5 Cell transfection

HOTAIR cDNAs were cloned to mammalian expression vector pcDNA3.1 (Invitrogen, USA). Small interfering RNA (siRNA) targeting HOTAIR (ie si‐HOTAIR) and controlled siRNA (si‐NC) were purchased from Ribobio (China). Gene Pharmaceutical cor. (China) designed and synthesized the miR‐17‐5p mimics, miR‐17‐5p inhibitor, and miR‐NC. According to the manufacturer's instructions (Invitrogen, USA), Lipofectamine 2000 was adopted for the transfection process.

### 2.6 Cell proliferation assay

Cells in the logarithmic phase of growth were taken out to prepare single‐cell suspension at the density of 1 × 10^5^ cells/mL, and cells were then inoculated into the 96‐well plates by 200 μL/well. The cell viability was detected by 20 μL MTT (5 mg/mL, Sigma, St. Louis, MO, USA) at the time points of 24, 48, 72, and 96 hours. Each well was added with 150 μl DMSO to slightly dissolve the crystallization at room temperature for 15 minutes. The absorbance of each well was measured by microplate reader at the wavelength of 490 nm (A). The proliferation rate of tumor cells was calculated in line with the formula as follows:$\frac{\mathrm{intervention}\;\;\mathrm{group}\;\;A\;\;490-\mathrm{blank}\;\;\mathrm{control}\;\;\mathrm{group}\;\;A\;\;490}{\mathrm{negative}\;\;\mathrm{control}\;\;A\;\;490-\mathrm{blank}\;\;\mathrm{control}\;\;\mathrm{group}\;\;A\;\;490}\times 100\%.$

### 2.7 Colony formation assay

About 500 cells were inoculated into the 6‐well plates, and they were cultivated in 5% CO_2_ at 37°C for 2 w. Subsequently, cells were fixed with methanol, and were stained with 0.1% crystal violet (Beyotime Institute of Biotechnology, Haimen, China). The colonies that included ≥450 cells were calculated.

### 2.8 Cell apoptotic assay

After 48‐h transfection, cells were washed with 0.01 mol/L PBS, and supernatants were discarded after 5‐minutes centrifugation at the speed of 1500 r/minute. At that time, cells were resuspended within the 1 × binding buffer, and their concentration was adjusted as 1 × 10^6^/mL. Meanwhile, 500 μL cell suspension, 5 μL Annexin V‐FITC, and 10 μL PI (Invitrogen, USA) were added to each tube, and they were mixed up. Under the conditions of room temperature with opaque background, cells were incubated for 10 minute, and were then evaluated by FACS flow cytometry (Becton Dickinson, Franklin Lakes, NJ, USA).

### 2.9 Drug susceptibility assay

Different concentrations of cisplatin (ie 0.00, 1.00, 2.50, 5.00, 7.50, 15.00, and 25.00 μg/mL), adriamycin (ie 0.00, 0.10, 0.50, 1.00, 2.50, 5.00, and 10.00 μg/mL), mitomycin (ie 0.00, 0.10, 0.50, 1.00, 2.50, 5.00, and 10.00 μg/mL), 5‐fluororacil (ie 0.00, 1.50, 3.00, 6.00, 12.00, 25.00, and 45.00 μg/mL) and SQFZ injection (ie 0.0, 10.0, 40.0, and 80.0 μg/mL) were, respectively, supplemented to 96‐well microplates for further 48 hours incubation. Then 20 μL MTT (5 mg/mL) was added to each well, and was then incubated for 4 hours. Next, the medium was removed, followed by addition of 100 μL DMSO. Finally, cells were measured with a microplate reader (Bio‐Rad, Hercules, CA, USA) at the wavelength of 540 nm, and the cells’ susceptibilities to drugs were assessed by comparing their 50% inhibitory concentration (IC50).

### 2.10 Western boltting

Total protein was extracted with RIPA lysis buffer (Beyotime Institute of Biotechnology, China). Proteins were separated using 12% SDS‐PAGE, and were then transferred onto polyvinylidene fluoride (PVDF) membranes. Subsequently, the PVDF membranes were blocked by utilizing Tween 20 (TBST) and Tris‐buffer saline that contained 5% skimmed milk powder at room temperature for 1.5 hours. The membranes were incubated with specific rabbit‐anti‐human PTEN monoclonal antibodies (1:1000, Abcam, Cambridge, MA, USA), rabbit anti‐human monoclonal antibodies for E‐cadherin, vimentin and N‐cadherin (1:1000, Proteintech, Manchester, UK) and rabbit anti‐human GAPDH monoclonal antibodies (1:1000, CST, Danvers, MA, USA) at 4°C for all night. After irrigating the membranes with TBST buffer for 3 times, and peroxidase‐labelled mouse anti‐rabbit secondary antibody (Zhongshan Bio‐Engineering, Guangzhou, China) were supplemented to the membrane for 2 hours incubation at room temperature. Lastly, enhanced chemiluminescence (ECL) reagents (Bio‐Rad, USA) were employed to make proteins visible, and Image J software (National Institutes of Health, Bethesda, MD, USA) was arranged to semi‐quantify the proteins.

### 2.11 Dual‐luciferase reporter gene assay

The HOTAIR fragments that included miR‐17‐5p binding sites were cloned into pmirGLO double oligosaccharide enzyme vector (Promega, Madison, WI, USA), and pmirGLO‐HOTAIR‐Wt reporter vector was constructed. The same HOTAIR fragments, except that their miR‐17‐5p binding sites were mutated, were named as pmirGLO‐HOTAIR‐Mut. When cell confluence reached 80%~90%, miR‐17‐5p and miR‐NC were, respectively, transfected into MKN28 cells that have been transfected with pmirGLO‐HOTAIR‐Wt or pmirGLO‐HOTAIR‐Mut. Similarly, the PTEN fragments that contained the binding sites for miR‐17‐5p were managed to construct PTEN‐Wt and PTEN‐Mut. And miR‐17‐5p and miR‐NC were also, respectively, transfected into MKN28 cells that have been transfected with pmirGLO‐HOTAIR‐Wt or pmirGLO‐HOTAIR‐Mut. About 48 hours later, luciferase activity was detected utilizing dual‐luciferase reporter gene assay system (Promega, Madison, WI, USA), and the relative activity was calculated as the ratio of firefly luciferase activity and renilla luciferase activity.

### 2.12 Statistical analyses

All the statistical analyses were carried out by means of SPSS 17.0 software, and images were processed with GraphPad 6.0 software. The measurement data [mean ± standard deviation (SD)] were compared with application of *t* test and one‐way ANOVA, and the enumeration data were analszed utilizing chi‐square test. Under the above conditions, a significant difference was indicated when *P *<* *0.05.

## 3 RESULTS

### 3.1 HOTAIR and miR‐17‐5p expressions within gastric cancer patients

**Figure 1:**
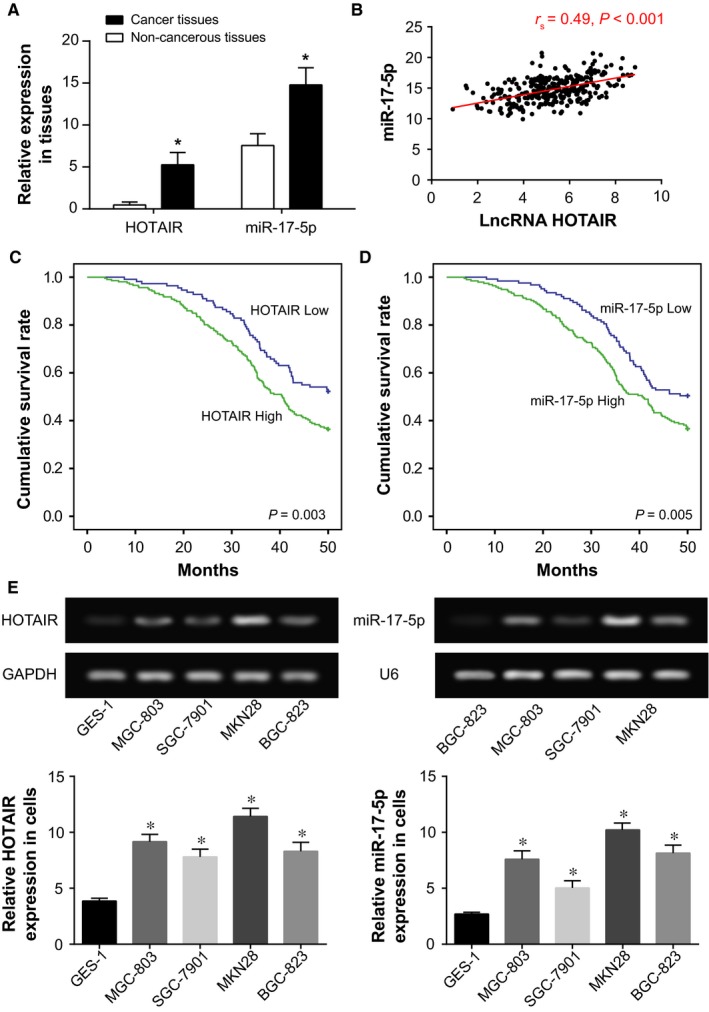
LncRNA HOTAIR, miR‐17‐5p expressions within gastric cancer tissues. A, The HOTAIR and miR‐17‐5p expressions were compared between gastric cancer tissues and non‐cancerous tissues. *P <* *0.05 when compared with non‐cancerous tissues. B, The HOTAIR expression was positively correlated with miR‐17‐5p expression within gastric cancer tissues. C, The Kaplan‐Meier curve was carried out among gastric cancer patients featured by highly and lowly expressed HOTAIR. D, The Kaplan‐Meier curve was carried out among gastric cancer patients featured by highly and lowly expressed miR‐17‐5p. E, The HOTAIR and miR‐17‐5p expressions were compared among BGC‐823, MGC‐803, SGC‐7901, MKN28, and GES‐1 cells. *P <* *0.05 when compared with GES‐1 cell

According to Figure 1A, HOTAIR and miR‐17‐5p expressions within gastric cancer tissues were, respectively, nearly 10‐ and 2‐folds of those within normal para‐carcinoma tissues (*P *<* *0.05). Furthermore, both higher HOTAIR and miR‐17‐5p expressions were discovered within gastric cancer patients who were poorly differentiated and at advanced AJCC stages (ie III+IV) (*P *<* *0.05) (Table 2). The cancer sufferers with lymph node metastasis and vessel invasion also more frequently possessed higher‐level HOTAIR and miR‐17‐5p expressions than ones whose lymph node metastasis and vessel invasion were unobserved (*P *<* *0.05). Interestingly, HOTAIR expression was positively correlated with miR‐17‐5p expression among the 317 gastric cancer patients investigated (r_s_ = 0.49; *P *<* *0.05) (Figure 1B). More than that, after excluding the influence of other parameters, higher HOTAIR expression, higher miR‐17‐5p expression, poor differentiation, advanced AJCC stage (III + IV) and presence of lymph node metastasis appeared as predictors for gastric cancer patients’ poor prognosis (*P *<* *0.05) (Table 3) (Figure 1C,D).

**Table 2:** Relation of HOTAIR and miR‐17‐5p expressions with clinical characteristics of gastric cancer patients

Characteristics	HOTAIR expression			miR‐17‐5p expression		
	Low (n = 111)	High (n = 206)	*P*‐value	Low (n = 123)	High (n = 194)	*P*‐value
Age (years)						
≤50	44	74	0.514	51	67	0.214
>50	67	132		72	127	
Gender						
Male	69	115		78	106	0.123
female	42	91	0.275	45	88	
HP						
Positive	63	124	0.553	78	109	0.202
Negative	48	82		45	85	
Size of tumor (cm)						
≥5 (large)	45	112	**0.019**	69	88	0.062
<5 (small)	66	94		54	106	
Grade of differentiation						
Poor and not	68	149	**0.043**	74	143	**0.011**
Well and moderate	43	57		49	51	
Location of tumor						
Cardia	23	40	0.552	25	38	0.203
Body	30	46		23	53	
Antrum	58	120		75	103	
AJCC stage						
III,IV	71	159	**0.012**	80	150	**0.017**
I,II	40	47		43	44	
Lymph node metastasis						
Present	86	180	**0.022**	96	170	**0.024**
Absent	25	26		27	24	
Vessel invasion						
Positive	29	98	**<0.001**	38	89	**0.008**
Negative	82	108		85	105	

The bold value indicated a significant results with a *P* value <* *0.05.

**Table 3:** Effects of HOTAIR and miR‐17‐5p expressions, as well as clinical characteristics on the survival rates of gastric cancer patients

Characteristics	Univariate analysis			Multivariate analysis		
	Hazard ratio	95% CI	*P* value	Hazard ratio	95% CI	*P* value
HOTAIR expression						
Low vs High	0.39	0.24‐0.63	**<0.001**	0.49	0.29‐0.83	**0.008**
miR‐17‐5p expression						
Low vs High	0.43	0.27‐0.69	**<0.001**	0.52	0.31‐0.87	**0.012**
Age (years)						
≤50 vs >50	1.09	0.69‐1.73	0.723	1.12	0.67‐1.88	0.667
Gender						
Male vs Female	1.19	0.76‐1.86	0.461	1.30	0.79‐2.16	0.302
HP						
Positive vs Negative	1.2	0.77‐1.89	0.424	1.45	0.87‐2.40	0.154
Size of tumor (cm)						
≥5(large) vs <5(small)	0.99	0.64‐1.55	0.977	1.15	0.70‐1.91	0.582
Grade of differentiation						
Poor and not vs Well and moderate	2.46	1.52‐3.99	**<0.001**	1.87	1.09‐3.20	**0.022**
Location of tumor						
Cardia vs Body	0.72	0.37‐1.43	0.349	0.61	0.29‐1.28	0.186
Cardia vs Antrum	0.85	0.45‐1.52	0.854	0.66	0.35‐1.24	0.196
AJCC stage						
III,IV vs I,II	2.56	1.54‐4.24	**<0.001**	1.91	1.10‐3.34	**0.023**
Lymph node metastasis						
Present vs Absent	3.04	1.63‐5.69	**<0.001**	2.30	1.13‐4.69	**0.021**
Vessel invasion						
Positive vs Negative	2.34	1.46‐3.76	**<0.001**	1.67	0.99‐2.82	0.055

The bold value indicated a significant results with a *P* value <* *0.05.

### 3.2 Effect of SQFZ on gastric cancer cell lines’ chemo‐sensitivities

**Figure 2:**
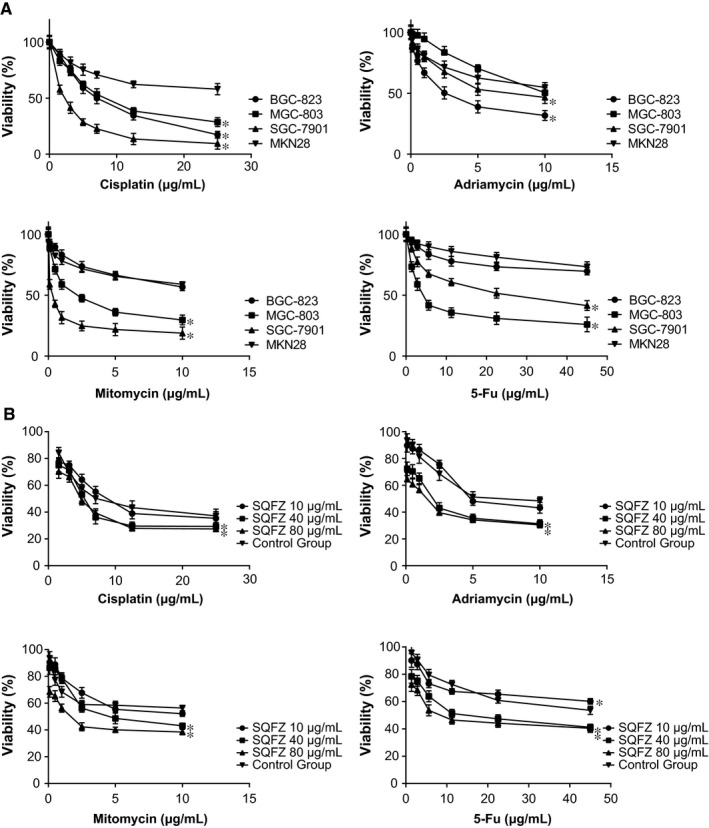
Selection of the gastric cancer cell line that was most sensitive to chemo‐drugs (i.e cisplatin, adriamycin, mitomycin, and 5‐fluororacil) and tradition Chinese medicine (SQFZ: Shenqifuzheng injection). A, The viabilities of BGC‐823, MGC‐803, SGC‐7901, and MKN28 cells were compared regarding their response to various concentrations of cisplatin, adriamycin, mitomycin, and 5‐fluororacil. *P <* *0.05 when compared with MKN28. B, The viabilities of BGC‐823, MGC‐803, SGC‐7901, and MKN28 cells were compared concerning their responses to SQFZ injection *P <* *0.05 when compared with control

Among the studied gastric cancer cell lines (ie BGC‐823, MGC‐803, SGC‐7901, and MKN28), HOTAIR and miR‐175‐5p displayed the highest expressions within MKN28 (all *P *<* *0.05) (Figure 1E). In addition, with increasing concentration of the added chemotherapeutic drugs, the cancer cells all presented a downward tendency in terms of their activity (*P *<* *0.05) (Figure 2A). Among them, MKN28 topped regarding resistance to cisplatin, adriamycin, mitomycin, and 5‐fluororacil (*P *<* *0.05). BGC‐823 came at the second place in its resistance to adriamycin and 5‐fluororacil (*P *<* *0.05), while MGC‐803 was next only to MKN28 considering resistance to cisplatin and mitomycin (*P *<* *0.05). From the above, MKN28 cell line was managed for the following experiments, and it was revealed that SQFZ could relieve the resistance of MKN28 cell line to chemotherapeutic stress, considering cisplatin, adriamycin, mitomycin, and 5‐fluororacil (all *P *<* *0.05) (Figure 2B).

### 3.3 SQFZ reversed the impacts of HOTAIR and miR‐17‐5p on gastric cancer cells’ chemosensitivity

**Figure 3:**
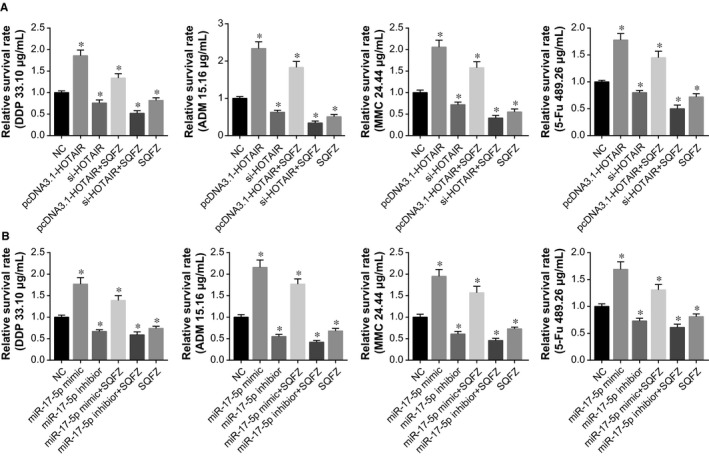
The relative survival rates of MKN28 cells were detected under the influences of HOTAIR or miR‐17‐5p combined with SQFZ (Shenqifuzheng) injection. A, The relative survival rates of MKN28 cells in response to chemo‐therapies were compared among pcDNA3.1‐HOTAIR, si‐HOTAIR, pcDNA3.1‐HOTAIR+SQFZ, si‐HOTAIR+SQFZ, SQFZ, and NC groups. B, The relative survival rates of MKN28 cells in response to chemo‐therapies were compared among miR‐17‐5p mimic, miR‐17‐5p inhibitor, miR‐17‐5p mimic+SQFZ, miR‐17‐5p inhibitor+SQFZ, SQFZ, and NC groups. *P <* *0.05 when compared with corresponding NC

Under the treatment of cisplatin (33.0 μg/mL), adriamycin (15.16 μg/mL), mitomycin (24.44 μg/mL), or 5‐fluororacil (489.26 μg/mL), MKN28 cell lines transfected with pcDNA3.1‐HOTAIR or miR‐17‐5p mimic were still accompanied with incremental activity in comparison to the control group (*P *<* *0.05). Nonetheless, transfection of si‐HOTAIR, miR‐17‐5p inhibitor, or pcDNA3.1‐PTEN into MKN28 cell line contributed to a tendency of decreased survival rate that was quite the opposite (*P *<* *0.05) (Figure 3). Intriguingly, simultaneous addition of SQFZ markedly down‐regulated the activity of MKN28 cell line (*P *<* *0.05) (Figure 3), suggesting that this TCM coordinated with si‐HOTAIR, miR‐17‐5p inhibitor, or pcDNA3.1‐PTEN to improve the chemosensitivity of MKN28 cell line.

### 3.4 Role of HOTAIR and miR‐17‐5p in regulating viability, proliferation, apoptosis EMT process of gastric cancer cells

**Figure 4:**
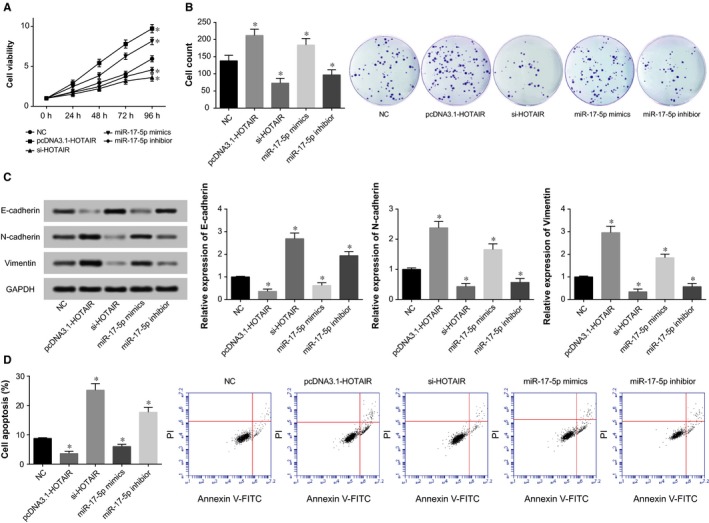
The MKN28 cells’ viability (A), proliferation (B), EMT‐specific proteins (i.e E‐cadherin, N‐cadherin and vimentin), (C) and apoptotic statuses (D) were compared after treatments with pcDNA3.1‐HOTAIR, si‐HOTAIR, miR‐17‐5p mimic, miR‐17‐5p inhibitor, and NC. EMT: epithelial‐mesenchymal transition; NC: negative control; *P <* *0.05 when compared with corresponding NC

In line with Figure 4, treatments with pcDNA3.1‐HOTAIR or miR‐17‐5p mimic triggered significantly incremental viability and proliferation, along with hindered apoptosis of MKN28 cell line (all *P *<* *0.05), yet MKN28 cell lines transfected with si‐HOTAIR and miR‐17‐5p inhibitor were detected with curbed viability and proliferation, along with increased apoptotic percentage (all *P *<* *0.05) (Figure 4). Regarding the EMT‐specific proteins, over‐expressed N‐cadherin and Vimentin, coupled with under‐expressed E‐cadherin were determined within both pcDNA3.1‐HOTAIR and miR‐17‐5p mimic groups (all *P *<* *0.05), and the si‐HOTAIR and miR‐17‐5p inhibitor groups exhibited a contrary trend (*P *<* *0.05).

### 3.5 The targeted relationships among HOTAIR, miR‐17‐5p, and PTEN

**Figure 5:**
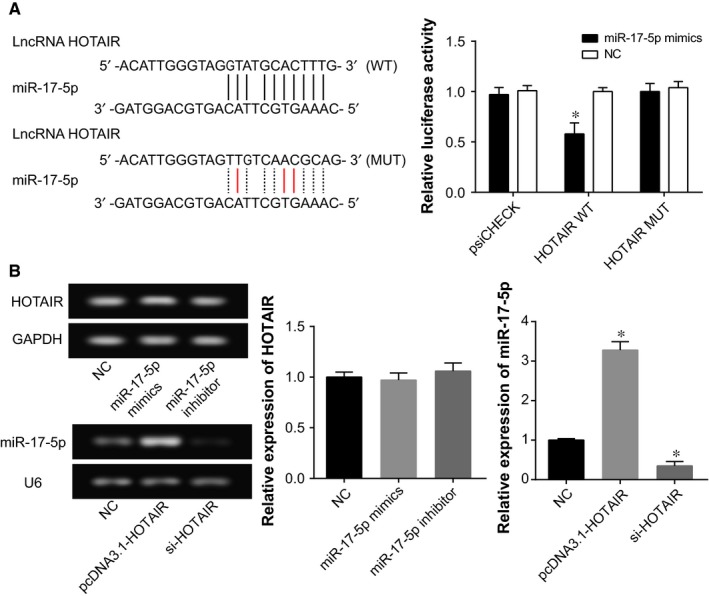
The correlation among HOTAIR and miR‐17‐5p within gastric cancer. (A) The luciferase activities were compared between miR‐17‐5p mimic+HOTAIR Wt and miR‐17‐5p mimic+HOTAIR Mut groups. *P <* *0.05 when compared with NC. (B) The effects of pcDNA3.1‐HOTAI, si‐HOTAIR on miR‐17‐5p expression and miR‐17‐5p mimic, miR‐17‐5p inhibitor on HOTAIR expression were assessed. *P <* *0.05 when compared with NC

The luciferase activity of pmirGLO‐HOTAIR‐Wt+miR‐17‐5p group was far below that of pmirGLO‐HOTAIR‐Wt+miR‐NC group (*P *<* *0.05), but pmirGLO‐HOTAIR‐Mut+miR‐17‐5p group possessed a luciferase enzyme activity that was no different from pmirGLO‐HOTAIR‐Wt+miR‐NC group (*P *>* *0.05) (Figure 5A). Moreover, altered miR‐17‐5p expressions within MKN28 cells were found to exert hardly any effects on the expressional level of HOTAIR, despite that largely modified miR‐17‐5p expressions were available when pcDNA3.1‐HOTAIR or si‐HOTAIR was supplemented (*P *>* *0.05) (Figure 5B).

**Figure 6:**
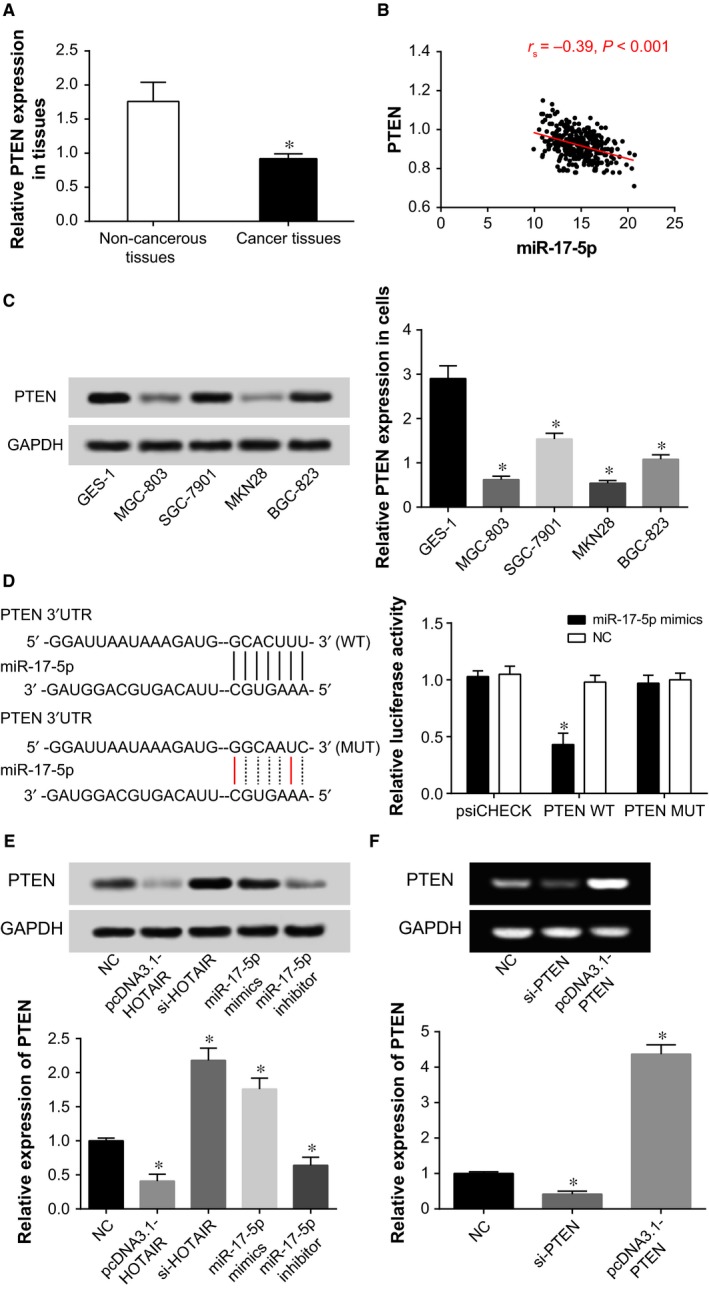
The PTEN expression was positively correlated with miR‐17‐5p expression within gastric cancer tissues (A, B). The PTEN expressions were compared among BGC‐823, MGC‐803, SGC‐7901, MKN28, and GES‐1 cells (C). *P <* *0.05 when compared with GES‐1 cell. The luciferase activities were compared between miR‐17‐5p mimic+PTEN Wt and miR‐17‐5p mimic+PTEN Mut groups (D). *P <* *0.05 when compared with NC. PTEN expressions were compared among pcDNA3.1‐HOTAIR, si‐HOTAIR, miR‐17‐5p mimic, miR‐17‐5p inhibitor, and NC groups (E). *P <* *0.05 when compared with NC. The effects of pcDNA3.1‐PTEN and si‐PTEN on PTEN expression were determined (F). *P <* *0.05 when compared with NC

Contrary to HOTAIR and miR‐17‐5p, PTEN expression within gastric cancer tissues achieved only half of that within para‐carcinoma tissues (*P *<* *0.05) (Figure 6A). Also PTEN expression was found to present a negative correlation with miR‐17‐5p expression (r_s_ = −0.39, *P *<* *0.05), and it was more lowly‐expressed within CRC cell lines (ie MKN28, MGC‐803, BGC‐823, and SGC‐7901) than within GES‐1 cell line (*P *<* *0.05) (Figure 6B,C). In an analogous manner, pmirGLO‐PTEN‐Wt co‐transfected with miR‐17‐5p presented the evidently reduced luciferase activity, when compared with pmirGLO‐PTEN‐Wt co‐transfected with miR‐NC (*P *<* *0.05). However, the detected luciferase activity of pmirGLO‐PTEN‐Mut+miR‐17‐5p group displayed no striking disagreement with that of pmirGLO‐PTEN‐Wt+miR‐NC group (*P *>* *0.05) (Figure 6D). A further phenomenon was found that the expressional level of PTEN was significantly changed within MKN28 cells after HOTAIR and miR‐17‐5p expressions were significantly up‐regulated or down‐regulated (*P *<* *0.05) (Figure 6E).

### 3.6 MiR‐17‐5p inhibited PTEN to modify viability, proliferation, apoptosis, and EMT process of gastric cancer cells

**Figure 7:**
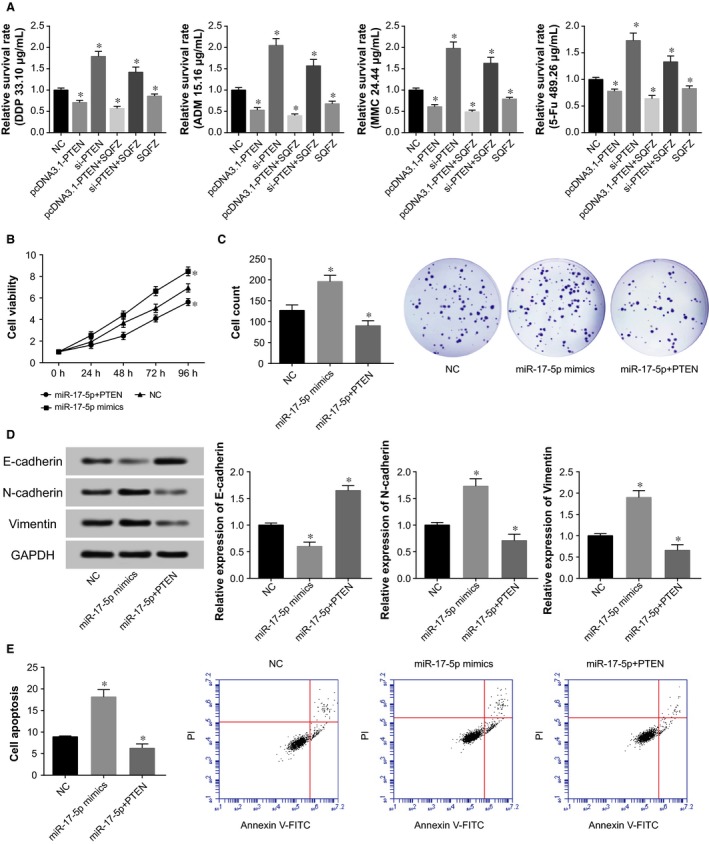
The role of PTEN in modifying gastric cancer cells’ responses to chemo‐radiotherapies. The relative survival rates of gastric cancer cells in the pcDNA3.1‐PTEN and si‐PTEN groups after treatments with chemo‐drugs (ie cisplatin, adriamycin, mitomycin, and 5‐fluororacil) and tradition Chinese medicine (SQFZ: Shenqifuzheng injection) (A). *P <* *0.05 when compared with NC. Regulatory effects of miR‐17‐5p on PTEN expression in regulating viability (B), proliferation (C), EMT‐specific proteins (D), and apoptosis (E) of MKN28 cell line. EMT: epithelial‐mesenchymal transition; *P <* *0.05 when compared with NC

After being treated with si‐PTEN or pcDNA3.1‐PTEN, MKN28 cells, separately, exhibited down‐regulated and up‐regulated PTEN expressions (*P *<* *0.05) (Figure 6F). Moreover, regarding treating cells with chemo‐drugs, MKN28 cell lines transfected with si‐PTEN were observed with elevated activity when compared with control group, yet pcDNA3.1‐PTEN group functioned reversely (*P *<* *0.05) (Figure 7A). It was manifested that miR‐17‐5p mimic group was associated with markedly higher viability and proliferative capacity of MKN28 cells in comparison to NC group (*P *<* *0.05). Addition of PTEN (ie miR‐17‐5p+PTEN group) seemed to interdict the impacts of miR‐17‐5p on MKN28 cells’ viability and proliferation (*P *<* *0.05) (Figure 7B,C). Concerning the EMT‐specific proteins, transfection with miR‐17‐5p mimic could induce increased N‐cadherin and vimentin expression, as well as decreased E‐cadherin expression (*P *<* *0.05) (Figure 7D). Nonetheless, extra addition of PTEN guided a converse trend (*P *<* *0.05). As for cell apoptosis, the apoptotic percentage of MKN28 cells in the miR‐17‐5p+PTEN group was significantly lower than that in the miR‐17‐5p mimic group (*P *<* *0.05) (Figure 7E).

## 4 DISCUSSION

Gastric cancer, a disorder with high degree of prevalence and mortality on a world scale, covered approximately 6.8% of total cancer incidence and 8.8% of cancer‐induced deaths.^26^ Multidrug resistance (MDR) would be generated under the stimulation of chemotherapies, thereby affecting the treatment effects for gastric cancer patients.^27^ In response, the current study attempted to deeply approach molecular mechanisms relevant to aggression and chemosensitivity of gastric cancer.

In recent years, lncRNAs and miRNAs have been increasingly explored in their involvement with pathogenesis of gastric cancer.^28^ The HOTAIR investigated here was observed to be aberrantly expressed within breast cancer,^9^ gastric cancer,^29^ colorectal cancer,^30^ and pancreatic cancer tissues.^31^ The expressional level of HOTAIR also appeared a biomarker regarding the stages, lymph node metastasis, vascular invasion, and survival rate of gastric cancer patients,^32^ and our study also confirmed this part (Figure 1) (Tables 2 and 3). More than that, highly expressed HOTAIR could promote gastric cancer cells’ anchorage‐independent growth and peritoneal diffusion.^33^ Simultaneously, HOTAIR could facilitate transformation of gastric cancer tissues from polarized epithelial cells to mobile mesenchymal cells,^34^ and also trigger abnormal expressions of genes relevant to EMT and cancer metastasis, including ICAM‐1, MMP1, MMP3, MMP9, and Snail.^34^ Correspondingly, knockout of HOTAIR could induce hindered EMT process, which could be reversed by addition of exogenous *Snail*.^35^, ^36^ Hence, it was well‐explained that HOTAIR might facilitate the progression of gastric cancer through mediation of the EMT process in this study (Figure 4). Furthermore, HOTAIR was documented to promote cancer cells’ resistance to platinum by up‐regulating the activity of PI3K/AKT signaling.^37^ Its high expression also boosted breast cancer cells’ resistance to tamoxifen via up‐regulation of ER receptor.^38^ With respect to gastric cancer, HOTAIR encouraged the cancer cells’ resistance to cisplatin by targeting miR‐126 and further elevating VEGFA and PIK3R2 expressions.^39^ It was also confirmed within this investigation that HOTAIR was responsible for improving gastric cancer cells’ resistance to chemo‐drugs, and this function was achieved probably through prohibiting apoptosis of cancer cells and encouraging viability and proliferation of cancer cells (Figures 3, 4).

As was documented before, HOTAIR could unite with miRNAs to improve or discourage the chemosensitivity of certain cancers.^39^ With patients suffering from non‐traumatic osteonecrosis of femoral head as the focus, HOTAIR was estimated to modulate proliferation and differentiation of osteoblasts through suppressing miR‐17‐5p/SMAD7 signaling.^40^ This targeted relationship between HOTAIR and miR‐17‐5p has also been verified within the studied gastric cancer cells by the luciferase reporter gene assay conducted here (Figure 5). Moreover, application of antago‐miR‐17‐5p could impede growth of chemo‐resistant neuroblastoma by blocking cell cycle and accelerating cell apoptosis.^41^ For another, elevated miR‐17‐5p expression could raise lung cancer cells’ resistance to docetaxel via prohibiting expression of autophagy‐relevant proteins (eg Beclin l).^42^ Consistently, it was explained in this study that miR‐17‐5p was subject to regulation of HOTAIR in its increasing cell viability and decreasing cell apoptosis, thereby down‐regulating gastric cancer cells’ chemosensitivity (Figures 3, 4).

This study also validated that miR‐17‐5p targeting PTEN was involved with altered growth and apoptosis of gastric cancer cells (Figure 6). It has been formerly documented that PTEN could serve as a biomarker for predicting progression and lymphatic metastasis of gastric cancer patients,^43^ and the gastric adenocarcinoma cells’ growth was obviously held up with elevated PTEN expression.^44^ The function of PTEN was also before deemed as the regulatory result of miR‐17‐5p, for instance, transfection of miR‐17‐5p inhibitor into tumor cells would boost PTEN and p21 expressions.^45^ More than that, transfection with PTEN was associated with augmented resistance of acute myeloid leukaemia (AML) or acute lymphoblastic leukaemia (ALL) cell lines to chemo‐therapies.^46^ To sum up, PTEN, regulated by upstream HOTAIR and miR‐17‐5p, was involved with modifying chemosensitivity of gastric cancer cells (Figures 6, 7).

Regarding SQFZ injection, its major ingredients consisted of astragalus and codonopsis. Treatment with astragaloside could partly reverse the chemo‐resistant tendency of gastric cancer cells line (ie BEL‐7402/FU).^47^ Moreover, the inhibitory impacts exerted by astragaloside on growth of gastric cancer cells were probably linked with its regulating COX‐2, VEGF, and PGE2 expressions.^48^ Mandy et al. documented that astragaloside could also induce apoptosis of colorectal cancer cells through triggering DNA fracture and chromatin condensation.^49^ Above all, astragaloside was biologically involved with pro‐immunity, anti‐inflammation, anti‐virus, anti‐tumor, radical scavenging, and so on.^50^, ^51^ Furthermore, multiple ingredients of flavonoids, one component of SQFZ, were also discovered to reverse multidrug resistance of tumor cells.^52^ Similar to the above publications, SQFZ injection was found to reverse the incremental chemo‐resistance caused by activated HOTAIR/miR‐17‐5p/PTEN axis (Figures 2, 3, 4, 5, 6, 7).

In summary, this research initially interpreted the inhibitory effects of HOTAIR/miR‐17‐5p/PTEN axis on chemosensitivity of gastric cancer cells, and drawn a conclusion that SQFZ injection could reverse this impact. However, the definite molecular mechanisms relevant to the functions of HOTAIR/miR‐17‐5p/PTEN axis and SQFZ injection demanded further studies. Nonetheless, merely in vivo studies were conducted, and experiments relevant to establishing mice models and clinical samples were left to be further explored, so that the mechanisms could be confirmed more deeply. For another, since the SQFZ injection adopted was only explicit in its several ingredients, extra ingredients should also be made certain. Through this approach, specific elements that played a dominant role could be extracted for developing doses in treating gastric cancer. What's more, the underlying mechanism within gastric cancer was intricate, and only an axis of the molecular signaling was focused on, so other crucial molecules should also be investigated in future.

## CONFLICT OF INTEREST

None.
